# High Amount of Physical Activity on Work Days Is Associated with More Intense Musculoskeletal Symptoms in Nurses: Seven-Day Observational Study

**DOI:** 10.3390/nursrep15050143

**Published:** 2025-04-27

**Authors:** Sarah Luna, David Douphrate, Byeong Yeob Choi, Bertha Flores, Rupal Patel, Lisa Pompeii

**Affiliations:** 1 School of Rehabilitation Sciences, University of the Incarnate Word, San Antonio, TX 78209, USA; 2 School of Public Health, Texas A & M University, College Station, TX 77843, USA; douphrate@tamu.edu; 3 Department of Population Health Sciences, Long School of Medicine, University of Texas Health Science Center at San Antonio, San Antonio, TX 78229, USA; choib@uthscsa.edu; 4 School of Nursing, University of Texas Health Science Center at San Antonio, San Antonio, TX 78229, USA; floresb2@uthscsa.edu; 5 School of Physical Therapy, Texas Woman’s University, Houston, TX 77030, USA; rpatel@twu.edu; 6 Division of Patient Services Research, Cincinnati Children’s Hospital, Cincinnati, OH 45229, USA; lisa.pompeii@cchmc.org

**Keywords:** musculoskeletal symptoms, physical activity, nurses, occupational health

## Abstract

**Background:** Musculoskeletal problems contribute to nurse attrition, which compromises patient safety and costs healthcare organizations millions of dollars. Recent research describes a physical activity paradox in which high amounts of work-related physical activity may be detrimental to health; however, there is a lack of evidence on the physical activity paradox with respect to musculoskeletal health in nurses. The purpose of this study was to examine the relationship between musculoskeletal symptoms (MSSs) and high amounts of physical activity at work in nurses. **Methods:** This was a 7-day observational design using direct measurement of physical activity and self-reported MSSs in nurses. Physical activity was measured in step counts using a wearable accelerometer and MSSs were reported using ecological momentary assessment. Step counts and MSSs were compared between work days and days off, and a regression model analyzed the combined effect of physical activity and work days on MSSs while controlling for age, exercise, and body mass index. **Results:** Musculoskeletal symptoms and step counts were significantly higher on work days compared to days off. Higher step counts on work days resulted in significantly higher expected MSS ratings than the same number of steps taken on a day off. **Conclusions:** This study supports the existence of a physical activity paradox in nurses with respect to MSSs. Understanding this paradox in the nursing workforce can translate to interventions that reduce the detrimental health effects of high levels of physical activity at work, which can minimize nurse attrition, improve patient outcomes, and reduce costs in healthcare organizations.

## 1. Introduction

Nurses are vulnerable to musculoskeletal symptoms (MSSs) due to the physical demands of their work [1,2], and MSSs are a common factor in nurses leaving the profession [3,4,5]. The positive general health benefits of physical activity are proven to be effective mechanisms to improve musculoskeletal health in healthcare workers [6,7], and the American Nurses Association (ANA) describes insufficient amounts of physical activity as one of the barriers to overall health in nurses [8]. Unfortunately, attempts to increase physical activity among nurses have not been successful, likely due to the demands of the job [9,10].

Physical activity is defined as body movement produced by skeletal muscles, whether for the purpose of leisure, transportation, or work [11]. Moderate and high-intensity physical activity promotes cardiopulmonary and cognitive function, maintains healthy body weight, prevents several types of cancers, and is effective in improving overall quality of life [11]. Nurses spend a majority of their shift standing or walking, which is categorized as light physical activity [12], but previous studies concluded that nurses are not consistently engaging in enough moderate- or high-intensity physical activity to achieve health benefits [13,14,15]. Brunet et al. attempted an online worksite intervention to increase physical activity in nurses and reported that nurses who worked in clinical roles had very little change in their physical activity [10]. Brunet and colleagues speculated that this limited change was likely due to fatigue and limited schedule flexibility compared to nurses in non-clinical roles such as a manager or researcher [10].

The evidence surrounding physical activity is beginning to acknowledge a difference between leisure-time physical activity and occupational physical activity [16]. The World Health Organization recommends being more physically active; however, this campaign focuses on total activity and does not differentiate between physical activity achieved during leisure time or at work [11]. Recent research discusses the existence of a ‘physical activity paradox’ in which not all physical activity is healthy [16,17,18,19]. A systematic literature review published in 2018 indicated that men who are engaged in intense physical activity at work have increased all-cause mortality compared to men whose jobs are less active [20]. A prospective study published in 2020 also supported the physical activity paradox using long-term sickness absence as an outcome measure [21]. In 929 workers who were monitored over 4 years, leisure physical activity was negatively associated with long-term sickness absence, and higher amounts of work physical activity were associated with a higher risk of long-term sickness absence [21].

The difference in health outcomes associated with leisure-time and occupational physical activity may be explained by the nature and characteristics of each [16]. Leisure-time physical activity is typically voluntary and completed in short bouts. Participants choose the type and intensity of the activity, and they can choose when to rest. Leisure-time physical activity is often followed by long recovery periods as well. Individuals do not have the same control over occupational physical activity, which is driven by the demands of the work tasks. Lack of autonomy or job control and fear of movement are associated with higher musculoskeletal symptoms in healthcare workers [22,23]. The duration of occupational physical activity may last the length of a work day, without the ability to self-determine rest periods or obtain sufficient recovery time before the next work shift.

Studying physical activity in nurses may support the physical activity paradox because nurses in clinical practice have high levels of physical activity at work [9]. A 2023 systematic literature review analyzed occupational physical activity and leisure-time physical activity in healthcare workers who were predominantly female [24]. The authors reported that high amounts of occupational physical activity were associated with low amounts of leisure-time physical activity, and preliminary study findings support the physical activity paradox with respect to cardiovascular health in nurses [24]. A 2021 study of nurses evaluated leisure-time physical activity, occupational physical activity, self-rated health, and the number of sick days taken from work [25]. High amounts of leisure-time physical activity had positive impacts on health and were associated with fewer sick days [25]. Conversely, high amounts of occupational physical activity were associated with poorer health and an increase in sickness absences [25]. Physical activity was reported using the International Physical Activity Questionnaire Short Form, which is a validated instrument; however, participants self-reported physical activity over the previous year [25]. The objective measurement of physical activity eliminates the possibility of bias introduced by self-reported instruments. A 2020 literature review called for a research agenda that better understands the physical activity paradox in predominantly female occupations in diverse global regions and uses objective measures of physical activity while distinguishing between physical activity at work and during leisure time [19].

Current research related to the physical activity paradox includes more male-dominated occupations than female occupations [16]. Existing studies primarily used self-reported measures of physical activity and outcomes such as self-rated health, cardiovascular health, mortality, and absence from work [16]. Merkus et al. studied the influence of occupational physical activity intensity on musculoskeletal pain in healthcare and construction workers by using heart rate to measure physical activity intensity and asking participants to recall their musculoskeletal pain intensity over the previous 4 weeks [17]. While not statistically significant, their findings suggested that higher amounts of physical activity intensity overall were associated with higher pain ratings [17]. The number of nurses included in the study was not reported, and recalling pain intensity over the previous 4 weeks introduced possible bias or inaccuracy [17].

This study builds on previous research by using a homogeneous occupational sample of only nurses in the United States, employing objective measurements of physical activity at work and during time off work and using real-time assessment of MSSs. Physical activity was measured in step counts using a validated wearable device (ActiGraph GT9X) [26,27,28,29,30,31,32] and musculoskeletal symptoms were measured using ecological momentary assessment to capture real-time data in the living and working environments. Ecological momentary assessment has been used to assess musculoskeletal symptoms, including pain and stiffness [33,34,35], and reduces the likelihood of recall bias while capturing the dynamic nature of musculoskeletal symptoms.

The purpose of this study was to examine the relationship between high levels of physical activity at work and MSSs among nurses. Specifically, this study tested the hypothesis that high amounts of physical activity at work are associated with higher MSS ratings compared to physical activity outside of work. The findings from this study contribute to the body of knowledge of the physical activity paradox in a predominantly female occupation. Understanding the relationship between MSSs and physical activity is important for guiding future occupational and public health interventions that can promote longevity in the nursing workforce.

## 2. Materials and Methods

### 2.1. Design

This study employed a cross-sectional, descriptive-correlational design. Physical activity was directly measured and MSSs were assessed using ecological momentary assessment over 7 consecutive days between October and December 2023.

### 2.2. Participants

This study was approved by the Institutional Review Board at the University of the Incarnate Word (2023-1397-EXP) and conducted in accordance with the ethical principles of the Declaration of Helsinki [36]. Nurses were recruited at the end of an online survey used in a prior study [37]. During an in-person meeting with the primary investigator, nurses were informed of the purpose, participant requirements, risks, and benefits of the study. After their questions were answered, nurses consented to participation by submitting their unique identifiers on an electronic form.

Participants were eligible if they were 18 to 65 years old and currently employed as a nurse with a primary role in direct patient care. Nurses only worked day shifts during the data collection period because disrupted sleep may influence MSSs. Nurses who had any kind of surgery in the past 3 months were excluded to prevent acute, post-operative symptoms from inflating MSS reports. Participants resided in the State of Texas so that the primary investigator, also based in Texas, could travel to meet with the nurses in person. All participants were compensated with a retail gift card.

Based on the parameter choices, for a desired power of 0.80, a Type I error rate of 0.05, and an effect size of 0.5, an estimated sample size of 29 participants was needed. The sample size was calculated using the UCSF Clinical & Translational Science Institute Sample Size Calculators [38].

### 2.3. Procedures

#### 2.3.1. Assessment of Physical Activity

Physical activity exposure was measured in daily step counts [39] because of their ease of interpretability and translation into an intervention in the future. Steps counts were tracked using wrist-worn accelerometers. Nurses wore an ActiGraph (GT9X link, ActiGraph, LLC., Pensacola, FL, USA) [40] on their non-dominant wrist for seven consecutive days. ActiGraph devices have good criterion validity for monitoring step counts when compared to manual step counts [26] and have been used in population health and clinical studies [27,28,29,30,31,32].

Since the ActiGraph collects data about its own movements, there is variability in the data collected from different body areas for different movements. The non-dominant wrist was selected as the ActiGraph location because it is validated and most accurately captures movements that contribute to nurses’ perceived physical work demands [41,42,43,44].

Nurses were asked to wear devices for 24 h per day to capture data during both sleep and waking hours and improve adherence to the research protocol by eliminating the need to remove and reapply the device at certain times of the day. Additionally, a 7-consecutive-day monitoring period is within the memory and battery life capability of the ActiGraph, which eliminated the need to charge the device. Nurses were instructed to continue their usual activities while wearing ActiGraphs; however, nurses were instructed to remove the device if wearing it could harm them or pose an injury or infection risk to their patients. Nurses were instructed to notify the primary investigator if they removed the device so that the time the device was not worn could be removed from the dataset.

ActiGraphs were initialized using the manufacturer’s default sampling rate of 30 Hz and programmed to begin recording data at 8 p.m. the night before Day 1 of the 7-day period. The display showed only the date and time to prevent feedback about activity levels from influencing nurses’ typical behavior.

Data were stored locally on the wearable device and then manually uploaded to ActiLife 6 data analysis software (ActiLife 6, ActiGraph, Pensacola, FL, USA) by the primary investigator at the end of the 7-day data collection period. Wear time was validated in ActiLife using the method described by Troiano et al. [45] and reconciled with any participant reports of removing the device. Raw accelerometry data were converted to step counts using the methods described by Hildebrand et al. [46] and van Hees et al. [47]. Step count data were exported from ActiLife and a new variable was created for cumulative steps. Cumulative steps were totaled at 6:00 a.m., 12:00 p.m., 6:00 p.m., and 9:00 p.m.

#### 2.3.2. Assessment of Musculoskeletal Symptoms

Musculoskeletal symptom ratings were assessed using ecological momentary assessment to capture real-time symptom location and intensity. Participants received a Qualtrics survey link (Qualtrics, Provo, UT, USA) via text message four times per day: 6:00 a.m., 12:00 p.m., 6:00 p.m., and 9:00 p.m. The survey prompted nurses to report MSS intensity using a 0 to 10 scale (0 = no symptoms, 10 = most severe).

Participants were asked to rate the severity of their musculoskeletal symptoms rather than their pain so that perceptions of discomfort, stiffness, and pain would be included. Musculoskeletal symptoms were operationally defined as any of the following sensations: pain, stiffness, soreness, or aches, and there was no differentiation between these symptoms in the analysis. Clinically, patients may not interpret discomfort or stiffness as pain. Discomfort was used to describe symptoms that “can be ignored” or are “not harmful” [48]. Stiffness was described as limited movement or mobility [48].

The survey sent at 6:00 a.m. asked participants to report whether they worked that day and, if it was a work day, what time their shift began. Shift end time was reported on the 9:00 p.m. survey, and nurses were also asked if they had had a lunch break and timely bathroom breaks during their shift. Because exercise contributes to activity metrics, nurses reported whether they exercised or not, and responses were included as a binary variable in the dataset. Exercise was defined as physical activity that was planned, structured, repetitive, and intended to improve or maintain physical fitness [49].

Musculoskeletal symptom ratings were aligned with the corresponding cumulative step counts. For example, the 12:00 p.m. MSS rating was aligned with the cumulative step count at 12:00 p.m. for that day. Shift length, in hours, was determined from shift start and end times.

Missing MSS ratings were determined by using an average of the ratings for that day. If physical activity was not captured (e.g., the nurse removed the ActiGraph), associated MSS ratings for that time were not included.

### 2.4. Statistical Analysis

Descriptive statistics were calculated for participant characteristics, work characteristics, physical activity, and MSS ratings. The distribution of MSS ratings was assessed for normality. Univariate analyses tested for associations between MSS symptom ratings, physical activity, and work days using a generalized linear mixed model regression with a log link and the participant identification number was included as a random effect to account for repeated measures within subjects. The same statistical procedure was used to evaluate the combined effect of physical activity and work days on MSSs while controlling for age, exercise, and body mass index (BMI). All statistical analyses were performed using SAS^®^ Studio software, Version 3.81. Copyright © 2012–2020 SAS Institute Inc., Cary, NC, USA.

## 3. Results

### 3.1. Participant Characteristics

Thirty-one nurses participated in the study, and a summary of their demographics is presented in Table 1. Each nurse participated for 5 to 7 days, resulting in 214 nurse-days in the dataset. Twenty-nine nurses participated for all 7 days, one nurse participated for 6 days, and one nurse participated for 5 days. Musculoskeletal symptoms were reported 4 times per day, resulting in a total N of 855 data points.

Thirty participants were female (97%) and one was male (3%). Sixteen nurses identified themselves as white, non-Hispanic (52%); thirteen were white, Hispanic (42%); one nurse was Black/African American (3%); and one nurse identified as some other race or ethnicity (3%). The average age of participants was 40.58 years (SD 9.82) and the average body mass index (BMI) was 29.09 (SD 5.79). Seven nurses (22.58%) had a BMI in the normal range, 13 were in the overweight category (41.19%), and 11 met the criteria for obesity (35.48%)

Out of 214 nurse-days, 119 (56%) were work days and 95 (44%) were days off. In total, 71% of participants (*n* = 22) worked in a hospital setting; 10% worked in outpatient care, urgent care, or a surgical center (*n* = 3); 13% worked in either home health or hospice; and another 6% (*n* = 2) included a school nurse and a transport nurse who were categorized as “other” in the Work Setting section of Table 1. The average shift length for nurses in this study was 10.73 h (SD 2.67). During the 7-day data collection period, 3 nurses worked 2 out of 7 days (10%), 11 nurses worked 3 days (36%), 8 worked 4 days (25%), 7 worked 5 days (23%), and 1 nurse worked all 7 days (3%). Nurses reported having a lunch break on 73.7% of the work days and no lunch break on 26.3%. Nurses were able to take a timely restroom break on 79.7% of their work days, and they reported having to delay restroom breaks 20.3% of the time.

When exercise was evaluated across the total number of nurse-days, nurses reported exercising on 33 of the 214 nurse-days (15%), with 5% being on work days and 10% being on days off. Nurses did not exercise on 80% of the nurse-days (*n* = 172), and they did not answer the exercise question 4% of the time (*n* = 9).

### 3.2. Musculoskeletal Symptoms

The distribution of MSS ratings was not normally distributed, with positive skewness and a high number of zero ratings. Because the variance for the distribution exceeded the mean (μ = 2.26, σ^2^ = 4.01), a negative binomial distribution was selected to account for overdispersion. The overall average MSS rating was 2.26 (SD = 1.69) and ranged from 0 to 10. Nurses reported no symptoms 27.85% of the time and, 72.19% of the time, nurses had symptoms of at least 1 out of 10.

**Figure 1:**
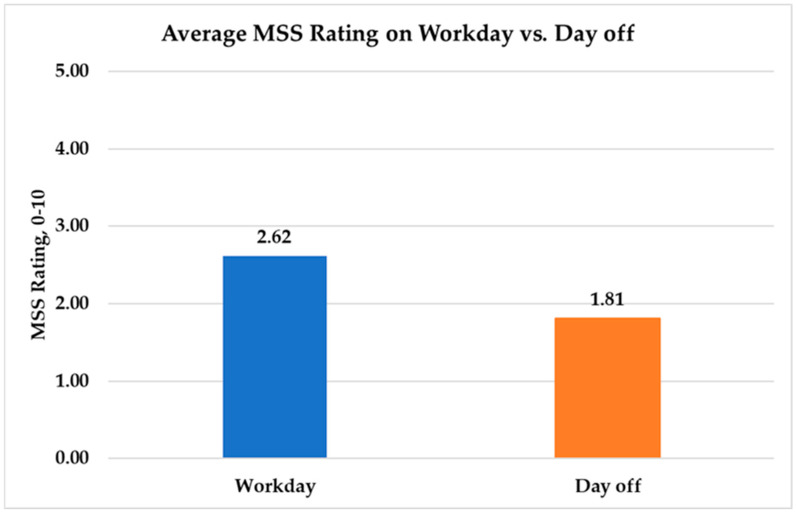
Average musculoskeletal symptom ratings on work days vs. days off.

Nurses rated MSSs significantly lower on days off compared to work days (Figure 1). The mean MSS rating on days off was 1.81 (SD = 0.69), while the mean MSS rating on work days was 2.62 (SD = 2.16) (t(823) = −4.76, *p* < 0.0001). Work days were associated with a 27.4% increase in expected MSS rating compared to days off (t(823) = 4.76, *p* < 0.0001).

**Figure 2:**
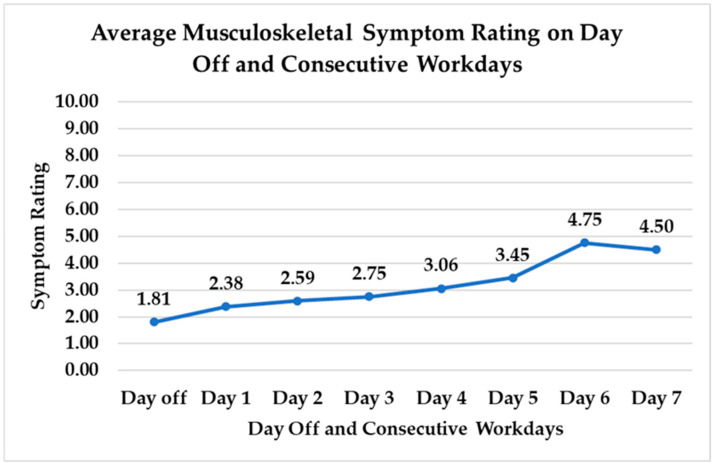
Average musculoskeletal symptom ratings on days off and consecutive work days.

Consecutive work days were also associated with higher MSS ratings, as demonstrated by a 6.69% increase in MSS ratings with each consecutive day of work (t(822) = 3.74, *p* = 0.0002) (Figure 2).

**Figure 3:**
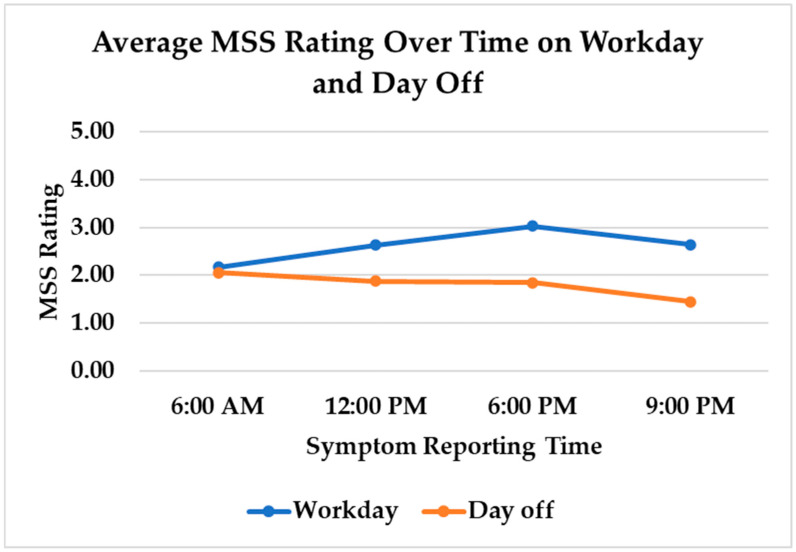
Average musculoskeletal symptom ratings over time on work days and days off.

When average MSS ratings were compared by time of day, average MSS ratings at 6:00 a.m. were similar on days off (M = 2.05, SD = 1.68) and work days (M = 2.17, SD = 2.01) (t(821) = 0.74, *p* = 0.46); however, there were significant differences at the other three time points. As pictured in Figure 3, average MSS ratings slightly decreased throughout the day on days off, while symptoms increased from 6:00 a.m. to 6:00 p.m. on work days and then decreased at 9:00 p.m. Musculoskeletal symptoms were significantly lower on days off at 12:00 p.m. (t(821) = −2.17, *p* = 0.03), 6:00 p.m. (t(821) −3.93, *p* < 0.0001), and 9:00 p.m. (t(821) = −4.51, *p* < 0.0001). MSS ratings were significantly higher at 6:00 p.m. than at any other time of day (t(823) = 2.70, *p* = 0.007).

Shift length, lunch breaks, and restroom breaks were significantly associated with MSSs. For each additional hour of shift length, the expected MSS rating increased by 4% (t(439) = 2.53, *p* = 0.01). Not having a lunch break during their shift was associated with a 22.8% increase in expected MSS rating (t(428) = −2.16, *p* = 0.03); however, not having a bathroom break was associated with a 26.3% lower expected MSS rating (t(2.15), *p* = 0.03).

Older age was associated with higher MSSs, as indicated by a 3.1% increase in expected MSS rating for each additional year of age (t(824) = 2.29, *p* = 0.02). Similarly, higher BMI was associated with higher MSS ratings. With each unit increase in BMI, the expected MSS rating increased by 6.9% (t(824) = 3.23, *p* = 0.001). Neither exercise nor work setting was associated with MSSs. The results of univariate analyses using generalized linear mixed models are shown in Table 2.

### 3.3. Physical Activity

**Figure 4:**
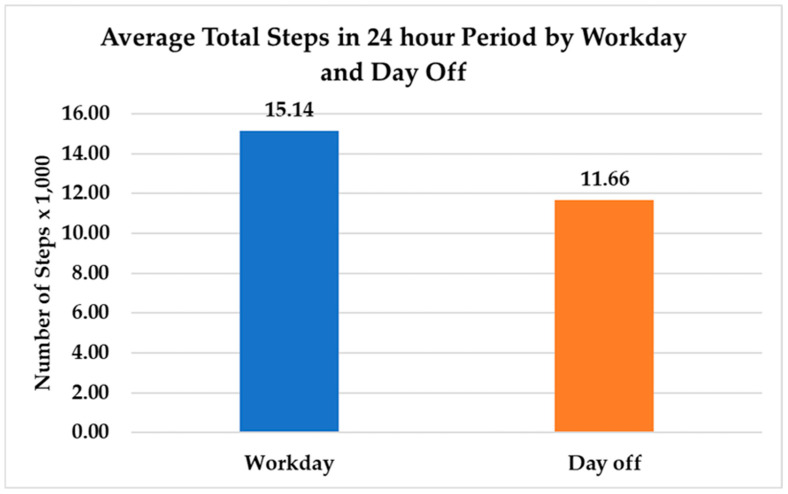
Average total steps in a 24 h period by work day and day off.

**Figure 5:**
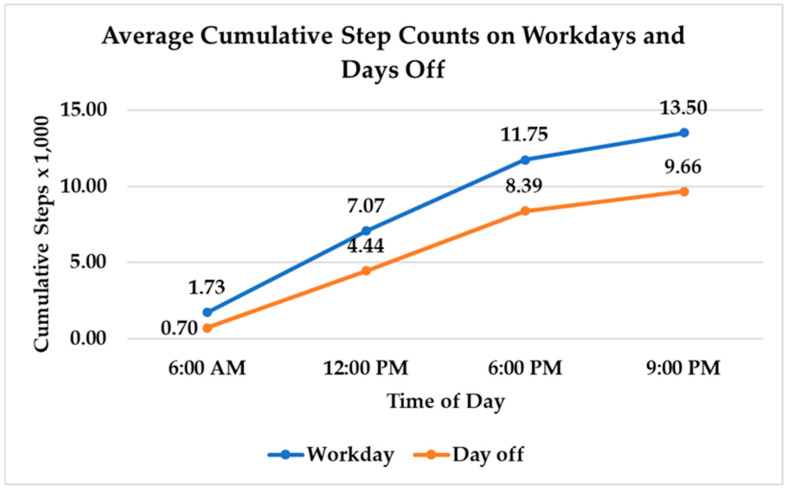
Average cumulative step counts on work days and days off.

The average number of steps on a work day was 15,170 (SD = 3500), which was significantly higher than the average number of steps on a day off (M = 11,660, SD = 3670) (t(823) = −14.68, *p* < 0.0001) (Figure 4). The total number of steps per day increased by 15% with each additional hour of shift length (t(439) = 3.78, *p* = 0.0002). Figure 5 displays the change in the average number of cumulative steps across times of day on work days and days off. The cumulative number of steps at the four MSS reporting times was significantly higher on work days compared to days off. Mean cumulative steps at 6:00 a.m. was 1730 (SD = 970) on work days and 700 on days off (SD = 520) (t(823) = −6.27, *p* < 0.0001); at 12:00 p.m., cumulative steps was 7070 (SD = 2160) on work days and 4440 (SD = 1850) on days off (t(823) = −4.94, *p* < 0.0001); at 6:00 p.m. cumulative step count was 11,750 (SD = 3930) on work days and 8390 (SD = 3620) on days off (t(823) = −0.34, *p* = 0.0001); and at 9:00 p.m., mean cumulative step count was 13,500 (SD = 5010) on work days and 9660 (SD = 4820) on days off (t(823) = −10.95, *p* < 0.0001). Higher cumulative step counts were associated with higher MSS ratings (t(823) = 2.58, *p* = 0.01).

### 3.4. Multivariable Model

A generalized linear mixed model regression analysis evaluated the combined effect of physical activity and work days on MSSs. Control variables included exercise, age, and BMI. Work setting was not included in the multivariable model because it was not significantly associated with step counts. While shift length, lunch breaks, and bathroom breaks were associated with MSSs, they were also strongly associated with step counts and work days. This created multicollinearity and resulted in an unstable statistical model.

When controlling for exercise, age, and BMI, the main effect of increased cumulative steps resulted in significantly lower MSS ratings (t(769) = −1.99, *p* = 0.05); however, when the main effect of cumulative steps was combined with the effect of work days, MSS ratings per 1000 cumulative steps were significantly higher on work days compared to the same 1000 steps taken on a day off (t(769) = 3.38), *p* = 0.0008) (Table 3). Higher BMI had a significant association with higher MSS ratings when all other variables were held constant (t(769) = 2.73, *p* = 0.007).

## 4. Discussion

The purpose of this study was to examine the relationship between MSSs in nurses with high amounts of physical activity at work. Nurses in this study were more physically active on work days compared to days off and they had significantly higher MSS ratings on work days compared to days off. Additionally, nurses reported significant increases in MSSs with each consecutive day of work.

When controlling for work days, exercise, age, and BMI, the effect of cumulative steps alone resulted in *lower* MSS ratings, which is consistent with previous findings about the benefit of total physical activity on the musculoskeletal system [50]. However, the interaction of cumulative steps and work day resulted in significantly *higher* expected MSS ratings for each 1000 steps taken on a work day compared to the same 1000 steps taken on a day off. This finding supports the existence of a physical activity paradox in the context of MSSs in nurses [18]. While research on the effect of the physical activity paradox on musculoskeletal symptoms is limited, the findings of this study are consistent with previous conclusions about the negative effects of high amounts of occupational physical activity on cardiovascular health, sick leave from work, and overall self-reported health [16,24,25]. The methods used in this study add to the body of knowledge about the physical activity paradox by using objective measurements of physical activity and real-time symptom reporting in living and working environments.

The global population takes an average of 5000 steps per day [39], and the average American takes 3000 to 4000 steps per day [51]. Previous research indicates that nurses have poorer health than the general population, in part due to a lack of physical activity [8]. The nurses in this study exceeded the recommended 10,000 steps per day by more than 30% [52]; however, they did not experience the health benefits of increased physical activity, as evidenced by MSSs and high average BMI. The average BMI (29.09) for the nurses in this study was higher than the national average for the American nursing workforce (27.58) [53]. The proportion of nurses who met the criteria for obesity (35.48%) was remarkably higher than the proportion for the global population (16%) [54], which may be explained by sampling nurses in Texas. The prevalence of obesity in the sample was similar to that of the state of Texas (35.7%) [55].

Previous studies describe the health benefits of leisure-time physical activity [16]; however, nurses participated in planned exercise outside of work on only 15.4% of the days during this study, and most of their steps were accumulated on work days. Physical activity at work does not appear to have the same health benefits as leisure-time physical activity due to less control over the type of activity and start/stop times [16]. Given the average shift length of 10.73 h, nurses likely did not have time for planned exercise on work days, and they needed their days off to recover from the physical demands of their job. Hence, increasing MSS ratings in nurses over consecutive work days is likely attributable to a lack of recovery time.

Multiple study limitations require cautious interpretation of these findings. First, data were collected from a relatively small sample for only one week, which is not long enough to capture personal and occupational trends that influence MSSs and physical activity. Second, this study was limited by the Healthy Worker Effect [56], a type of selection bias inherent in studying individuals who are healthy enough to remain in the workforce. Additionally, the convenience sample of nurses in Texas may not reflect global trends in physical activity or musculoskeletal health. While a sample of primarily females is expected when studying the nursing workforce, the findings cannot be confidently applied to nurses who are male.

ActiGraph placement at the wrist most accurately reflects nurses’ awkward upper extremity positions and whole-body movements; however, this location may overestimate physical activity by including seated upper extremity movements, such as those used during computer tasks. By aligning symptom reports with cumulative step counts at the same time of day, the data did not consider the possibility of the delayed onset of symptoms. Additionally, this study did not consider physical activity intensity levels, psychological factors, biomechanical loading, or the initial physical condition of the participant.

Continued research to address the health of the nursing workforce is critical because musculoskeletal problems and poor health are common reasons for nurse attrition [4,5,57], which compromises patient safety and has costly consequences for healthcare organizations [58]. Future research should use a longitudinal design with a larger sample size to capture personal and occupational trends that influence MSSs over several weeks or months. Additional information about physical activity intensity and biomechanical load on the musculoskeletal system would allow the alignment of physical demand levels with MSSs. Understanding activity intensity thresholds may offer more explanation about which intensity levels of occupational physical activity have a higher risk of negative health benefits. Additional information about occupational psychological factors and specific work units within the healthcare setting will provide a deeper understanding of relationships between MSSs, occupational physical activity, and mental health. Additional data on the initial physical condition and health status of the participants would allow a statistical model to control for comorbidities that may influence musculoskeletal symptoms. The findings from this study can inform translational research that addresses the high physical demands of a nurse’s role. Step counts may be used as a measure of job demand in future occupational health interventions. For example, wearable devices and ecological momentary assessment data can be used in interventional studies aimed at balancing workload with adequate recovery time.

Healthcare organization leaders and clinicians should apply research findings about the physical activity paradox to discussions about health and physical activity with employees and patients. Rather than asking about overall physical activity, clinicians should differentiate between leisure-time physical activity and occupational physical activity to obtain a more complete picture of the patient’s risk for MSSs and poor health. Healthcare organization leaders who attempt to promote physical activity among their employees will need to understand that occupational physical activity may be contributing to the poor health of the workforce.

## 5. Conclusions

This study explored the physical activity paradox in nurses, where high amounts of occupational physical activity are detrimental to musculoskeletal health. Nurses in this sample did not exercise outside of work consistently, which may have been due to long shift lengths and the need to use days off work to recover. Nurses in this study averaged over 10,000 steps per day; however, their activity was not associated with musculoskeletal health and weight benefits since the average BMI approached the obese category.

High amounts of physical activity on work days were associated with significantly higher MSSs than the same amount of physical activity on a day off. Musculoskeletal problems contribute to nurse attrition, which compromises patient safety and costs healthcare organizations millions of dollars. Future musculoskeletal interventions should focus on reducing job demand, as measured by physical activity at work, and increasing recovery time between shifts.

## Figures and Tables

**Table 1:** Sociodemographic and work-related variables.

Characteristics	N = 31	Percent
**Gender**		
Male	1	3.2
Female	30	96.8
**Race/Ethnicity**		
Black/African American	1	3.2
White, non-Hispanic	16	51.6
White, Hispanic	13	41.9
Other race/ethnicity	1	3.2
**Work setting**		
Hospital	22	71
Outpatient care, urgent care, surgery center	3	9.7
Home health	4	12.9
Other	2	6.5
**Days of Participation**		
5 days	1	3.2
6 days	1	3.2
7 days	29	93.6
**Work days**		
2–3 days	14	45.2
4–5 days	15	48.4
6–7 days	2	6.4
**Days worked (out of 214 nurse-days)**	**N = 214**	**Percent**
Work day	119	55.6
Day off	95	44.4
**Exercise (out of 214 nurse-days)**		
Exercised on work day	10	4.7
Exercised on day off	23	10.8
Did not exercise	172	80.4
Did not report	9	4.2
**Days with exercise**	**N = 29**	**Percent**
None	14	48.3
1–2	8	27.6
3–4	7	24.1

**Table 2:** Univariate associations with musculoskeletal symptom ratings.

Variables	Estimate	DF	t Value	*p*
**Cumulative Steps**	0.0117	823	2.58	0.01 *
**Time of Day**				
6:00 a.m.	−0.0863	823	−1.61	0.11
12:00 p.m.	0.0230	823	0.44	0.66
6:00 p.m.	0.1372	823	2.70	0.007 **
9:00 p.m.	−0.0842	823	1.54	0.12
**Day Off vs. Work Day**	0.2420	823	4.76	<0.0001 **
**Consecutive Work Days**	0.0669	822	3.76	0.0002 **
**Shift Length**	0.0389	439	2.53	0.01 *
**Lunch Break**	−0.2050	428	−2.16	0.03 *
**Bathroom Break**	0.2336	428	2.15	0.03 *
**Setting**				
Hospital	−0.2704	824	−0.90	0.37
Outpatient Care, Urgent Care, Surgical Center	−0.0246	824	−0.05	0.96
Home Health, Hospice	0.5483	824	1.38	0.17
Other	−0.0835	824	−0.15	0.88
**Age**	0.0303	823	2.29	0.02 *
**BMI**	0.0668	824	3.23	0.001 **
**Exercise**	−0.0974	773	−1.28	0.20

* *p* < 0.05. ** *p* < 0.01.

**Table 3:** Regression model explaining associations between musculoskeletal symptoms, step counts, and work days.

Variable	Parameter Estimate	Standard Error	DF	t Value	*p*
Intercept	−2.02	0.70	28	−2.90	0.01
Cumulative Steps ^a^	−0.02	0.01	769	−1.99	0.05 *
Work Day	−0.01	0.09	769	−0.14	0.89
Day Off ^b^	0.00				
Cumulative Steps ^a^ on Work Day	0.03	0.01	769	3.38	0.001 **
Cumulative Steps ^a^ on Day Off ^b^	0.00				
Exercise	0.00	0.08	769	−0.06	0.95
No Exercise ^b^	0.00				
Age	0.02	0.01	769	1.79	0.07
BMI	0.06	0.02	769	2.73	0.007 **

^a^ Cumulative steps × 1000. ^b^ Reference value. * *p* < 0.05. ** *p* < 0.01.

## Data Availability

The datasets presented in this article are available upon request from the corresponding author.

## Public Involvement Statement

No public involvement in any aspect of this research.

## Guidelines and Standards Statement

This manuscript was drafted using the STROBE guidelines for cross-sectional studies by von Elm, E.; Altman, D.G.; Egger, M.; Pocock, S.J.; Gotzsche, P.C.; and Vandenbroucke, J.P.: Strengthening the Reporting of Observational Studies in Epidemiology (STROBE) Statement: guidelines for reporting observational studies (2007).

## Abbreviations

The following abbreviations are used in this manuscript:

MSS	Musculoskeletal Symptom
ANA	American Nurses Association
LLC	Limited Liability Corporation
M	Mean
SD	Standard Deviation
